# Cd^2+^ removal efficiency of activated carbon from *Prosopis juliflora*: Optimization of preparation parameters by the Box-Behnken Design of Response Surface Methodology

**DOI:** 10.1016/j.heliyon.2024.e31357

**Published:** 2024-05-16

**Authors:** Gilbert C. Chintokoma, Yonas Chebude, Shimelis K. Kassahun

**Affiliations:** a African Centre of Excellence for Water Management, College of Natural and Computational Sciences, Addis Ababa University, P.O. Box 1176, Addis Ababa, Ethiopia; b Department of Chemistry, College of Natural and Computational Sciences, Addis Ababa University, P.O. Box 1176, Addis Ababa, Ethiopia; c School of Chemical and Bio Engineering, Addis Ababa University, P.O. Box 1176, Addis Ababa, Ethiopia

**Keywords:** Cadmium, Characterization, Optimization, *Prosopis juliflora*, Preparation parameters

## Abstract

The study focuses on the preparation of activated carbon from *Prosopis juliflora* (PJAC) wood by pyrolysis and chemical activation. The objective is to assess its effectiveness as an adsorbent for synthesizing a composite adsorbent coating (CAC) for Cadmium (Cd^2+^) removal from aqueous solution. The effect of preparation factors related to Cd^2+^ removal efficiency was assessed. The Design of Experiments (DoE) for the adsorption of Cd^2+^ on the PJAC were done using the Box-Behnken Design (BBD) of the Response Surface Methodology (RSM) (Design Expert software version 11). The influence of impregnation ratio (IR), carbonization time (t), and carbonization temperature (T) on the Cd (II) percent (%) removal was evaluated. The response surface graphs in 3D were also generated for the response variable, and the higher R^2^ coefficient values were fitted into the polynomial quadric model. The results indicated that all the variable preparation factors were significant in the Cd^2+^ removal by PJAC with carbonization temperature being the most significant. At the optimum conditions i.e. impregnation ratio (1.8), carbonization temperature (595 °C) and carbonization time (174 min), the model predicted a 99.9 % Cd^2+^ removal efficiency while the adsorption experiment obtained a 96.7 % removal efficiency, respectively. Later, the morphological and chemical properties of the PJAC prepared with optimal parameters were analyzed using different characterization techniques including S_BET_, SEM-EDX, pH_PZC_, FTIR and XRD. The SEM images revealed a rough and porous morphological surface with an S_BET_ of 600.4 m^2^/g and a near neutral pH_PZC_ of 6.92. The XRD pattern indicated the crystalline nature of the prepared adsorbent. The pre and post adsorption FTIR spectrum of the PJAC demonstrated a distinct difference with the latter showing a reduction in peak intensity and height. These results underpin the potential of utilizing invasive plants like *Prosopis Juliflora* as adsorbents for heavy metal removal.

## 1 Introduction

The growth of industry has resulted in the increasing availability of heavy metals in industrial effluent [1,2]. Industry effluents are the primary source of water pollution issues, mainly because the industrial sector utilizes a tremendous quantity of water while concurrently producing a substantial amount of highly polluted wastewater [3]. The issue of heavy metal pollution has become a major global environmental concern, primarily because of its substantial toxicity and ability to accumulate in the food chain, even at trace amounts [4]. Toxic heavy metals are a hazard to both animals and people and the environment in general due to their non-biodegradability, extended biological half lifetimes, and ability to collect in various body regions [5]. One of the heavy metals, Cadmium, has been linked to many symptoms, including vitamin D insufficiency, respiratory conditions, and gastrointestinal issues leading to erythrocyte depletion. These factors can hinder the optimal functioning of calcium in the bodies of both humans and animals [5,6].

Treatment technologies play a crucial role wastewater treatment and subsequent reuse. These encompass primary, secondary, and tertiary treatment methods depending on purpose [7]. In certain instances, multiple treatment methods are utilized in combination with others [8]. Adsorption is the most cost-effective technique for removing contaminants in water and wastewater due to its affordability, ease of operation and design [[9], [10], [11]]. Adsorption as a technique for heavy metal removal produces high quality effluent and the adsorbent can be regenerated making it economical. With regards to heavy metals adsorption from water and wastewater, the removal mechanism is quite intricate as it depends on factors such as the adsorbent used as well as the heavy metals being removed [12].

Recent research on adsorption has mainly concentrated on utilizing affordable environmentally friendly adsorbents, mainly because the extensive utilization of commercial activated carbon for water and waste treatment is not economically viable, primarily due to its exorbitant initial purchase and operational expenses [13]. One such low cost adsorbent is the invasive *Prosopis Juliflora* [14,15]. Originally from Peru, *Prosopis Juliflora,* was initially introduced in Africa (mainly arid and semi-arid) and other regions mainly for land reclamation [16]. Unfortunately, due of its invasive character attributed to several factors, the fast spread of *P. juliflora* is regarded as a danger to pastoralist livelihoods in the environment [17]. Periodically, the adverse effects on human livelihoods and the environment are intensifying at an alarming rate; therefore, it is critical to develop more efficient management strategies that can substantially mitigate negative consequences while maximizing positive ones. The utilization of *Prosopis Juliflora* as an adsorbent for water and wastewater treatment offers an alternative opportunity for managing and eradicating this detrimental invasive species.

Studies undertaken in Ethiopia and other areas has consistently found that the disadvantages of the *Prosopis Juliflora* plant outweigh its benefits [[14], [15], [16],[18], [19], [20]]. The research has suggested the sustainable use of the invasive plant species as a method to entirely eradicate it. Several researches have been conducted to investigate *Prosopis juliflora* plant's potential as an adsorbent for pollution abatement [[21], [22], [23], [24], [25], [26], [27], [28]]. The low moisture, high carbon, and lignocellulose contents as determined by [[29], [30], [31]] qualifies *Prosopis Juliflora* as a suitable biosorbent. These two factors played a role in the decision to use *Prosopis Juliflora* wood as biosorbent material for this study.

The Brunauer-Emmett-Teller (BET) surface area (S_BET_) and adsorption capacity (q_e_) of activated carbon are the most crucial properties in adsorption [32]. These properties are affected by the activated carbon's preparation parameters including the carbonization time, chemical impregnation ratio and carbonization temperature among others. These parameters influence the prepared activated carbon's surface properties and pore development. The three aforementioned parameters are essential preparatory factors for obtaining activated carbon as they influence the formation and distribution of a particular surface area and pore diameter respectively [33].

This study utilized *Prosopis Juliflora* wood material to prepare activated carbon (PJAC), which was used as an adsorbent for the removal of Cd^2+^ from aqueous solution. Specifically, the study examined and optimized the effect of impregnation ratio (IR) with Zinc Chloride (ZnCl_2_), carbonization temperature (T) and carbonization time (t) on PJAC Cd^2+^ removal efficiency. Using Box-Behnken design (BBD) of the Response Surface Methodology (RSM) (Design Expert version 11) the optimal preparation parameters were determined. The Cd^2+^ removal efficiency was then connected to the three variables using empirical models. The activated carbon derived from these optimal conditions was then characterized using; X-ray diffraction (XRD), Brunauer-Emmett-Teller (BET) surface area analysis (S_BET_), Scanning Electron Microscopy (SEM), Fourier Transform Infrared (FT-IR) spectrophotometer, and Point of Zero Charge (pH_PZC_). To the best of the authors knowledge, there is no prior research that has been done regarding the application BBD of the RSM as an optimization tool for combined chemical (ZnCl_2_) and thermal activation processes to prepare activated carbon from *Prosopis Juliflora* wood for Cd^2+^ adsorption from aqueous solutions.

## 2 Methods and materials

### 2.1 *Prosopis juliflora* wood powdered activated carbon preparation

This work was done at Addis Ababa University's (AAU) College of Natural and Computational Sciences' (CNCS) Core Water Laboratory. All of the solvents and chemicals utilized were of the reagent grade. The wood from *Prosopis juliflora* was collected at Kurkura, Awash in Ethiopia. A blade was employed to sever the spikes and outer branches. The biomass was extensively cleansed with tap-water to eliminate dirt and followed by rinsing with deionized (DI) water. The cleaned wood biomass was chopped into smaller pieces (≤2 cm) and was then dried in the sun for 7-days.

The desiccated precursor was saturated with a heated ZnCl_2_ solution at various impregnation ratios (1, 1.5, 2.0) for a duration of 2 h, and subsequently immersed in the very same ZnCl_2_ solution for 24 h. After 24 h, excess ZnCl_2_ solution was poured out and the biomass was allowed to air dry after being thoroughly washed with deionized water. Subsequently, the impregnated biomass was introduced into a muffle furnace and subjected to carbonization at various temperatures (namely, 400 °C, 500 °C, 600 °C) for varying durations (60–180 min) in order to remove the volatile components. The carbon was further cleansed with deionized water, dehydrated in a 40 °C oven for 24 h, pulverized, and then subjected to a temperature of 800 °C in a muffle furnace for 120 min, with a consistent heating rate of 10 °C per minute. This process was carried out to enhance the activated carbon's porosity as well as surface area. The prepared activated carbon was then sieved using a 150-μm screen and was placed in sealed containers for analysis and further adsorption experiments.

### 2.2 PJAC characterisation

Physico-chemical characteristics examined on the synthesized activated carbon were; BET surface area (S_BET_), surface charge (point of zero charge (pH_PZC_)), functional groups (Fourier-transform infrared spectroscopy (FTIR)), surface morphology (Scanning Electron Microscopy (SEM)), and the crystalline/amorphous structure (X-ray diffraction (XRD)) of the adsorbent. The adsorbent characterization was made only on the PJAC produced under the optimal preparation parameters. BET technique was used for PJAC surface area determination. To calculate the specific surface area (m^2^/g), measurements of N_2_ adsorption/desorption isotherms were conducted at temperature of −196 °C using a Micromeritics equipment (ASAP 2420). Prior to registering the isotherms, the samples underwent outgassing at a temperature of 150 °C for a duration of 16 h under high vacuum. The PJAC morphology was measured using SEM (Hitachi TM 1000). The pH drift method was used for PJAC pH_PZC_ determination (Eutech Instruments PH 700 series pH meter). The initial pH values of the 0.1 M NaCl electrolyte were set from 3 to 12 while employing 0.1 M HCL and a 0.1 M NaOH solutions respectively for pH adjustment. The functional groups of the adsorbent's surface were analyzed using FT-IR measurements. The measurements were conducted at room temperature using a Spectrum 65 FT-IR instrument in KBr pellets (PerkinElmer). The FTIR spectrum was performed between the wavelength range of 4000 to 400 cm^−1^. The structural arrangement of the PJAC pre and post Cd^2+^ adsorption was examined using XRD (Rigaku MiniFlex 600 Benchtop) which was carried out in scan range of 5°–80° in 2 theta angles, 40 kV, 44 mA and of 10 deg/min scan speed. On the other hand, chemical composition of *Prosopis Juliflora* in terms of ultimate (Carbon (C), Hydrogen (H), and Nitrogen (N) and other elements such as Sulphur (S) and Oxygen (O)) (the primary components), and proximate analysis (i.e. moisture, ash content and volatile material) respectively were sourced from literature [[29], [30], [31]].

### 2.3 Design of batch adsorption experiments (DoE)

The BBD of the RSM was employed in the Design of Experiments (DoE). The initial step of the BBD's DoE consisted of identifying the independent variables [34]; thus the impregnation ratio (*X*_*1*_), carbonization temperature (*X*_*2*_), and carbonization time (*X*_*3*_) and their respective ranges (limits). The parameters were coded at 3 levels: −1(low); 0(center); +1(high) (Table 1).

**Table 1:** Levels and experimental range of independent variables for the batch adsorption experiments.

Variable	Unit	Factors	Levels		
			−1	0	+1
**Impregnation Ratio (IR)**	–	*X*_*1*_	1	1.5	2
**Carbonization Temperature (T)**	°C	*X*_*2*_	400	500	600
**Carbonization Time (t)**	Min	*X*_*3*_	60	120	180

In this study, a 3-factorial and a 3-level BBD with 3 replicas at the central point was used to conduct 15 trials for response surface modelling. The levels and limitations of the independent factors were established using values derived from a variety of sources, including literature and pilot/preliminary tests. Equation (1) below was utilized to code the predetermined independent variables.

(1) $$X_{i}=\frac{X_{I\;}-X_{0}}{\Delta X}$$

*X*_*i*_ represents the dimensionless coded value of the of *i*th variable, *X*_*0*_ corresponds to the value of the variable in the center whereas Δ*X* denotes the step change. The model terms were generated and the experimental data was fitted using Equation (2). It is a model that is based on empirical data and uses a second-order polynomial equation [35].

(2) ***Y*** = *b*_0_ + ∑ b_ii_X_i_ + ∑ b_ii_X _i_^2^ + ∑ b_ij_X_i_X_j_

Where, the response variable ***Y*** represents Cd^2+^ reduction. The constant coefficients *b*_0_ is the intercept whereas b_i_, b_ii_ and b_ij_ correspond to the linear, quadratic and interaction terms respectively. The three independent variables (impregnation ratio (IR), carbonization time (t) and carbonization temperature (T)) are represented by X_i_ and X_j_.

### 2.4 Cadmium adsorption experiments and removal efficiency

Cd^2+^ stock solution with 1000 mg/L concentration was prepared by dissolving 17.9 g of the cadmium chloride hydrate (CdCl_2_⋅H_2_O) in 1 L of de-ionized water using a 1 L volumetric flask. For all the batch adsorption experiments, 100 mg/L Cd^2+^ concentration (made by dissolving 100 ml of the prepared 1000 mg/L stock solution into a 1 L volumetric flask) was used.

In each of the 15 batch tests, 25 ml of a solution containing 100 mg/L of Cd^2+^ was placed into a 150 ml Erlenmeyer flask. Then, 0.5 g of each of the prepared PJAC was added to the flask. The pH of the solution was adjusted to 8.0. The mixtures were agitated concurrently using a mechanical shaker at a speed of 200 revolutions per minute and a temperature of 25 °C for a duration of 30 min.

Following adsorption, the samples underwent centrifugation at a speed of 3600 revolutions per minute for a duration of 20 min. Inductively Coupled Plasma - Optical Emission Spectrometry (ICP-OES) (Agilent 5800) was used to measure the Cd^2+^ residual concentrations in the supernatant solutions. Equation (3) was utilized to calculate the Cd^2+^ percentage removal efficiency, R, (in %) while Equation (4) was used to determine the PJAC adsorption capacity, q, (in mg/g).

(3) $$R=\frac{C_{i}-C_{f}}{C_{i}}\times 100$$

(4) $$q_{e}=\frac{\left(C_{i}-\;C_{f\;}\right)}{m}V$$

Where ***R*** is the Cd^2+^ percent removal (%) efficiency, ***q***_***e***_ is the PJAC Cd^2+^ adsorption capacity (mgg^−1^) at equilibrium, ***C***_***i***_ and ***C***_***f***_ are the initial and final Cd^2+^ concentrations (mg/L) respectively, V corresponds to adsorbate volume (L) and ***m*** corresponds to mass of PJAC used (g).

### 2.5 Statistical analysis and model fitting

To obtain the prediction models for the removal efficiencies the BBD was used. Using the optimal settings, the model's ability to predict the optimal response value for Cd^2+^ percent removal efficiency was assessed (Table 4). Model suitability was examined and applied to the experimental data to describe whether or not the predicted model would generate incorrect or ambiguous results. This present work included many statistical tests, including sequential model (sum of squares), lack of fit tests, and model summary, to verify the adequacy of the model in demonstrating the best Cd^2+^ percent removal efficiency (%). Using Design Expert version 11, regression analysis was conducted to fit mathematical models to the experimental data in order to identify the best region for the responses under investigation. The statistical significance of the interactions was done using Analysis of Variance (ANOVA) (Table 4).

## 3 Results & discussion

### 3.1 Adsorbent proximate and ultimate analysis

Carbon samples’ proximate analysis provides information about the moisture and volatile matter, fixed carbon and ash content. On the other hand composition of C, H, N as well as other elements such as S and O (the primary components) in weight percentage is revealed upon doing an ultimate analysis of the carbon samples [13]. Table 2 summarizes the proximate and ultimate analysis of *Prosopis Juliflora* from previous studies [[29], [30], [31]]. The table demonstrates that the low ash and high carbon content of *Prosopis Juliflora* make it a promising candidate for usage as a raw material for activated carbon preparation for use as an adsorbent [36,37].

**Table 2:** *Prosopis juliflora* Proximate and ultimate analysis.

Analysis type	Biomass (weight %)		Activated carbon (weight %)
Proximate analysis			
*Reference*	[29]	[30]	
Moisture	11.5	10.85	2.98
Volatile matter	71	72.83	17.54
Fixed carbon	15.19	14.47	78.56
Ash	2.22	1.85	0.92
***Ultimate analysis***			
*Reference*	[31]	[30]	
Carbon	47.16	45.20	79.81
Hydrogen	5.81	5.58	2.48
Nitrogen	0.42	0.65	0.61
Oxygen	46.41	51.50	17.04
Sulphur	0.20	0.07	0.61

### 3.2 Statistical analysis

#### 3.2.1 ANOVA, model fitting and model diagnostic test

The correlations between PJAC preparation variables and Cd^2+^ removal efficiency as the response values was developed by the BBD. Table 3 displays the comprehensive design matrices along with the response values gleaned from the experimental results. Runs 10–12 were undertaken at the center point to assess the experimental error and also check study results’ repeatability. Using Equation (3), the Cd^2+^ percentage removal efficiency (%) ranged from 41.25 % to 99.82 %.

**Table 3:** BBD experimental design setup and results for the synthesis of the PJAC.

Run	Impregnation Ratio, IR, (*X*_*1*_)	Carbonization Temperature, T, (°C) (*X*_*2*_)	Carbonization Time, t, (min) (*X*_*3*_)	Initial Cd^2+^ Concentration (mg/L)	Final Cd^2+^ Concentration (mg/L)	Cd^2+^ Percent Removal Efficiency (%)
1	1.5	500	120	100	40.43	59.57
2	1.5	600	180	100	58.5	41.5
3	1	500	60	100	28	72
4	1	600	120	100	37.82	62.18
5	2	400	120	100	17.45	82.55
6	1.5	400	180	100	1.62	98.38
7	1	400	120	100	49.25	50.75
8	1	500	180	100	58.75	41.25
9	1.5	500	120	100	41.43	58.57
10	2	500	180	100	49.25	50.75
11	1.5	500	120	100	40.75	59.25
12	1.5	400	60	100	42.81	57.19
13	2	600	120	100	50.69	49.31
14	2	500	60	100	38.02	61.98
15	1.5	600	60	100	0.18	99.82

**Table 4:** The ANOVA and regression analysis findings for the quadratic model's response surface for Cd^2+^ % removal efficiency by PJAC adsorption.

Source	Sum of Squares (SS)	Degree of Freedom (df)	Mean Square	F-value	p-value	
**Model**	4516.53	9	501.84	43.33	0.0003	significant
A-Impregnation Ratio	152.69	1	152.69	13.18	0.0150	
B-Carbonization Temperature	1323.81	1	1323.81	114.31	0.0001	
C-Carbonization Time	245.98	1	245.98	21.24	0.0058	
AB	935.75	1	935.75	80.80	0.0003	
AC	0.0000	1	0.0000	2.159E-06	0.9989	
BC	8.44	1	8.44	0.7287	0.4323	
A^2^	323.48	1	323.48	27.93	0.0032	
B^2^	1380.34	1	1380.34	119.19	0.0001	
C^2^	49.53	1	49.53	4.28	0.0935	
**Residual**	57.91	5	11.58			
Lack of Fit	18.58	3	6.19	0.3151	0.8182	not significant
Pure Error	39.32	2	19.66			
**Corrected Total SS**	4574.43	14				

R^2^ = 0.9873; adjusted R^2^ = 0.9646; predicted R^2^ = 0.9157; adequate precision = 21.0418.

The study assessed the coefficients of the model for constant terms, quadratic effects, cubic effects, and interaction effects. ANOVA and regression analysis findings for the quadratic model of the response surface for Cd^2+^ removal efficiency using PJAC adsorption are displayed in Table 4. The *p-value* evaluates each coefficient's statistical significance and the intensity of the interaction among the factors, with effects below 0.05 being deemed significant [35]. As the level of significance increases, the relationship between observed and predicted values also increases [38]. The model's F-Value of 43.33 showed that the model was significant (*p* = *0.0003*) implying that at-least one of the variables in the model significantly influences Cd^2+^ adsorption by PJAC. For the Lack of Fit test, the F value of 0.32 and *p-value* of 0.8182 indicates that the Lack of Fit is not significant compared to pure error and also that it is not statistically significant for the experimental settings respectively. This suggests that the model is sufficient [39]. Table 4 shows that of all the individual parameters under study, carbonization temperature (T) was the most significant preparation parameter (Sum of Squares (SS) = 1323.8, *p-value* = *0.0001*), followed by carbonization time (SS = 245.98, *p-value* = *0.0058*) and the least significant parameter was the impregnation ratio (IR) (SS = 152.69, *p-value* = *0.015*).

The high regression coefficient (R^2^ = 0.9873) showcases the model's ability to forecast response in the experimental input data. The observed 21.04 adequate precision indicates an acceptable and adequate signal-to-noise ratio (i.e. >4). Again the adjusted R^2^ of 0.9122 is lower than the R^2^ value (0.9873), indicating that “the additional input variables are or could not add value to the model” [40]. The Predicted R^2^ (0.9157) and the adjusted R^2^ (0.9646) exhibit a satisfactory level of concordance as the difference between them is less than 0.2. According to the measured R^2^ values, the PJAC adsorption process was satisfactorily explained by the regression models. Consequently, the model developed in this work was deemed adequate for predicting Cd^2+^ removal efficiency from aqueous medium.

**Fig. 1:**
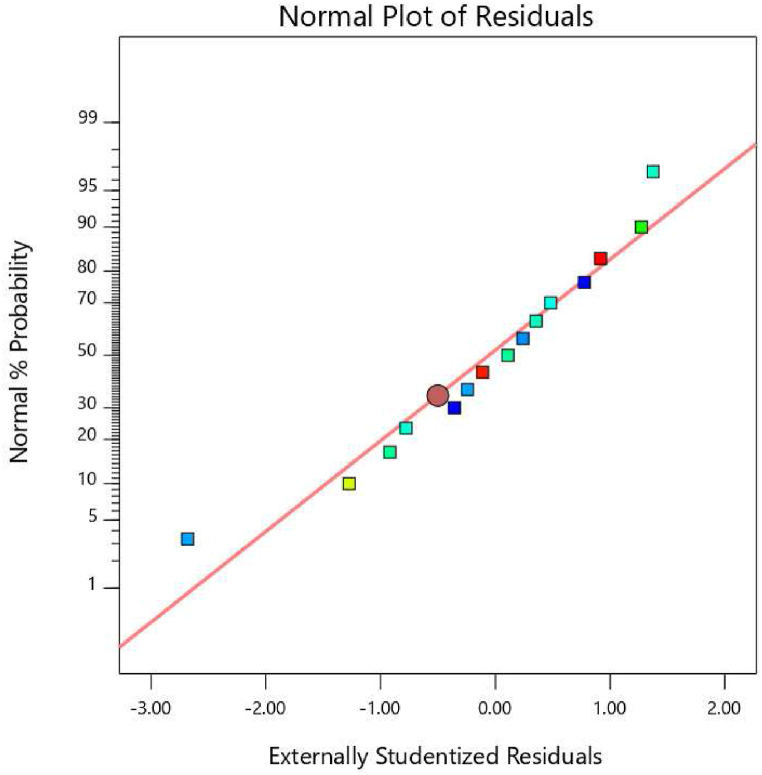
Normal probability plots PJAC adsorption process.

Fig. 1 depicts the standard probability curve of residues. The output may be inferred from the fact that the model is deemed acceptable due to the occurrence of a fairly linear relationship among the points in the graph.

**Fig. 2:**
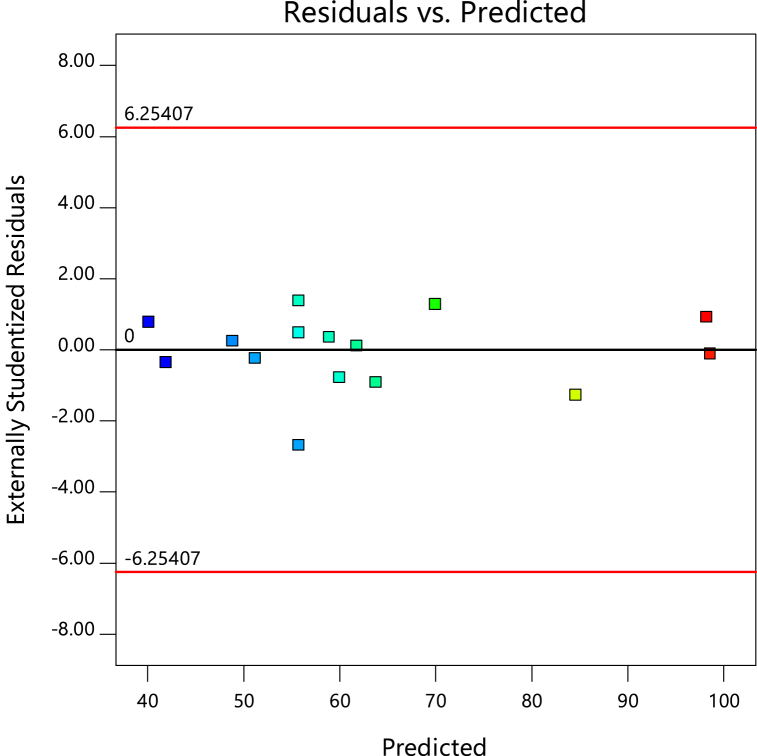
Residuals and predicted results plots for Cd2+ removal by PJAC adsorption process.

**Fig. 3:**
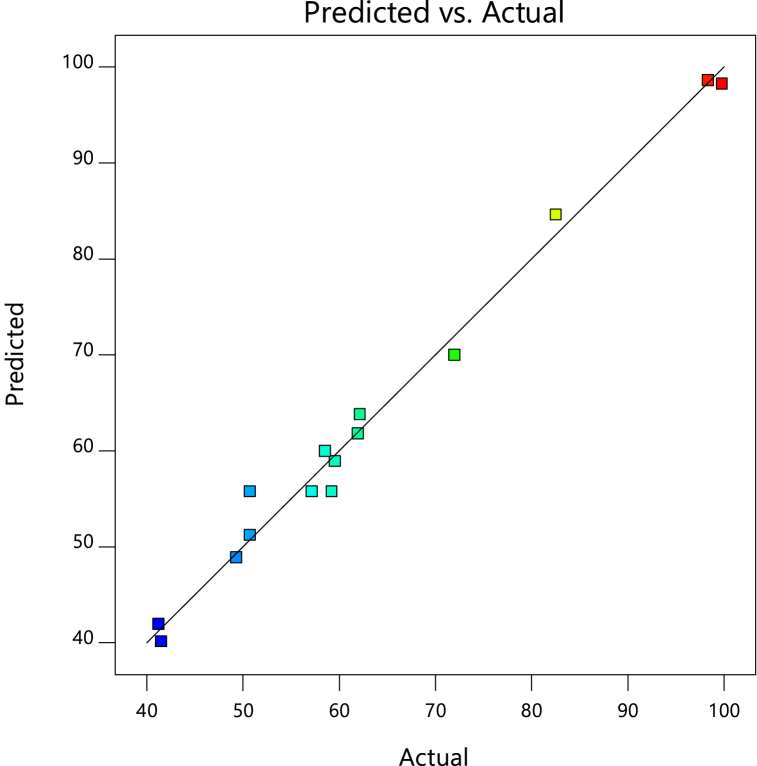
Predicted and actual results plots for Cd^2+^ removal by PJAC adsorption process.

The model's correctness was determined by examining the experimental diagnostic plots and outcomes of the model (Fig. 2, Fig. 3). With regards to actual residues against the fit plot, for a model to be reliable; a model must not exhibit any sequence of rising or falling point patterns in the real residues versus the fitted values [35,41]. The close alignment between residuals and predicted Cd^2+^ removal in Fig. 2 corroborates the results presented in Table 4.

The predicted and actual graphs in Fig. 3 further support the strong correlation between the projected and actual results. This indicates that the model was capable of making accurate predictions within the constraints of the set of the preparation parameters [35]. It can, therefore, be concluded that the response surface's quadratic model developed in this work accurately describes the experimental data of Cd^2+^ removal efficiency with preparation factors in PJAC adsorption.

#### 3.2.2 Model equations based on coded and actual factors

A quadratic model was chosen based on the recommendation of BBD of Design Expert for the PJAC Cd^2+^ adsorption from an aqueous solution. Basing on the sequential model sum of squares (SS), the models were chosen by considering the highest order polynomials with significant additional terms and avoiding any model aliasing. The equations for the percentage removal efficiency of Cd (II) are expressed by second order polynomial equations (5), (6), representing the coded and actual factors, respectively.

(5) $$Coded\;factors\;Cd\left(II\right)Removal\;Efficiency\;\left(\%\right)=55.73+4.37A+12.86B+5.55C+15.3AB+0.002AC+1.45BC-9.36A^{2}+19.34B^{2}+3.66C^{2}$$

(6) $$Actual\;factors\;Cd\left(II\right)Removal\;Efficiency\;\left(\%\right)=624.9650-31.9025IR-2.2928T-0.2729t+0.3059IR*Temp+0.0001IR*t+0.0002T*t-37.44{IR}^{2}+0.0019T^{2}+0.001t^{2}$$

Where *A*, *B*, and *C* represent the coded values and *IR*, *T*, and *t* represent the actual values for impregnation ratio, the carbonization temperature (in degrees Celsius), and the carbonization time (in minutes) respectively.

### 3.3 Effect of preparation parameters

**Fig. 4:**
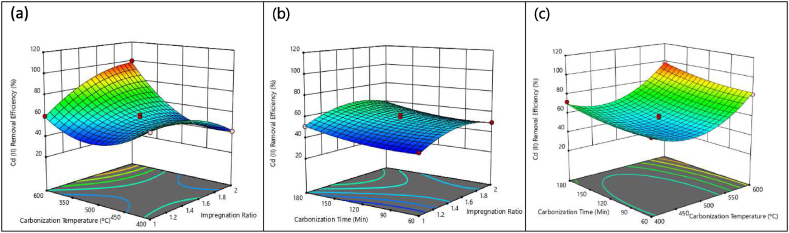
Three-dimensional response surface plots for PJAC Cd^2+^ removal efficiency (%); (a) effect of IR and T, (t = 120 min); (b) effect of IR and t, (T = 500 °C); (c) effect of t and T, (IR = 1.5).

Fig. 4 depicts the 3-dimensional response surfaces showing the effect of PJAC preparation parameters on Cd^2+^ removal efficiency (%). The effect of varying impregnation ratio (IR) and carbonization temperature (T) on Cd^2+^ removal efficiency (%) by the PJAC was evaluated while keeping the carbonization time (t) constant at 120 min (Fig. 4a). As shown in Fig. 4a Cd^2+^ removal efficiency increases as the impregnation ratio increases. However, once the impregnation ratio exceeds 1.7, the removal efficiency begins to decline. Notably, as the proportion of chemicals increases, there is a gradual improvement in the metal ion removal efficiency [42]. The improvement in the removal efficiency as the IR increases up to 1.7 is due to an increase in the specific surface area as well as total pore volume [43].

Again, from the FTIR spectra (Fig. 6), it is shown that the activated carbon contains phenol groups, which have acidic characteristics. Consequently, the Cl from ZnCl_2_ interacts with the H from phenol. As a result, the oxygen from phenol which is now hydrogen atom free would introduce a pair of electrons into the vacant orbital of Zn [44]. As explained by both [45,46], ZnCl_2_ possesses two Cl; therefore, it can react with two phenol-containing structures to create cross-links. Since the ZnCl_2_ forms cross-links and creates a stiff matrix, raising the ZnCl_2_ ratio would result in higher Cd^2+^ removal efficiency until IR 1.7 as shown in Fig. 4a. However, as the impregnation ratio is increased past 1.7, there is a decrease in the efficiency of Cd^2+^ removal. This is due to the excessive activation caused by the higher proportion of zinc chloride, which alters the structure of activated carbon. As a result, the pores may connect or disappear [33], which lowers the efficiency of Cd^2+^ removal as observed.

The high Cd^2+^ removal efficiencies with increasing carbonization temperature was attributed to increase in both micro and mesopores. A porous structure develops because at higher carbonization temperatures there is release of more volatile chemicals [45]. Temperatures above 450 °C can cause an increase in porosity due to the development of tar, which occurs when lignocellulosic material is treated with acid [47]. [43] found that at temperatures exceeding 450 °C; an increase in IR also increases the micro and mesopore volume [43]. The pore-widening process might be considerably accelerated by excess ZnCl_2_, which would result in the development of a mesoporous structure [48].

Fig. 4b displays the three-dimensional response surface that illustrates how the Cd^2+^ removal efficiency is affected by the impregnation ratio (IR) and carbonization time (t) (T = 500 °C), while Fig. 4c shows the interactive influence of carbonization temperature (T) and carbonization time (t) on Cd^2+^ removal efficiency (IR = 1.5). Fig. 4b indicates that the effect of carbonization time (t) on Cd^2+^ removal efficiency was not significant (p > 0.05), despite the varying impregnation ratios (IR). From Fig. 4c, it is evident that the Cd^2+^ removal efficiencies vary significantly (p˂0.05) with carbonization time (t) at different carbonization temperatures (T). When the carbonization time (t) is short (≤1.5 h) and the carbonization temperature (T) is low (≤500 °C), the recorded Cd^2+^ removal efficiencies are relatively low. This is likely due to insufficient time for the complete development of porosity in the char structure [46].

### 3.4 Process optimization

The process optimization was conducted using BBD of the RSM with the objective of maximizing the Cd^2+^ removal efficiency (%). The influencing preparation parameters were all set in range; impregnation ratio (1–2), carbonization temperature (400–600 **°**C) and carbonization time (60–180 min). Out of the 53 solutions suggested, optimum adsorption conditions (Desirability = 1) were obtained and selected at Impregnation ratio 1.77, carbonization temperature 595**°**C and carbonization time 173.6 min for 99.9 % Cd^2+^ removal (Table 5). The function's strong desirability suggests that it may accurately describe the experimental model and conditions.

**Table 5:** Some of the solutions suggested and selected for preparation parameters optimization tests for Cd^2+^ removal by *PJAC*.

Number	Impregnation Ratio	Carbonization Temperature (°C)	Carbonization Time (minutes)	Cd^2+^ Removal Efficiency (%)	Desirability	
**1**	**1.771**	**595.132**	**173.628**	**99.935**	**1.000**	**Selected**
2	1.618	598.577	177.291	99.134	1.000	
3	1.622	599.934	176.062	99.725	1.000	
4	1.698	594.349	174.096	98.125	0.971	
5	1.940	599.460	144.679	98.979	0.977	
6	1.726	598.102	169.944	98.986	0.989	
7	1.791	597.774	157.278	98.464	0.969	
8	1.967	599.164	178.218	98.659	0.986	
9	1.978	599.587	135.367	97.783	0.953	
10	1.948	599.904	146.795	99.676	1.000	

### 3.5 Adsorbent characterization

After optimization, fresh activated carbon was prepared with the determined optimized preparation parameters values and then this prepared PJAC was subjected to the following characterisation.

#### 3.5.1 SEM-EDX and S_BET_ analysis

**Fig. 5:**
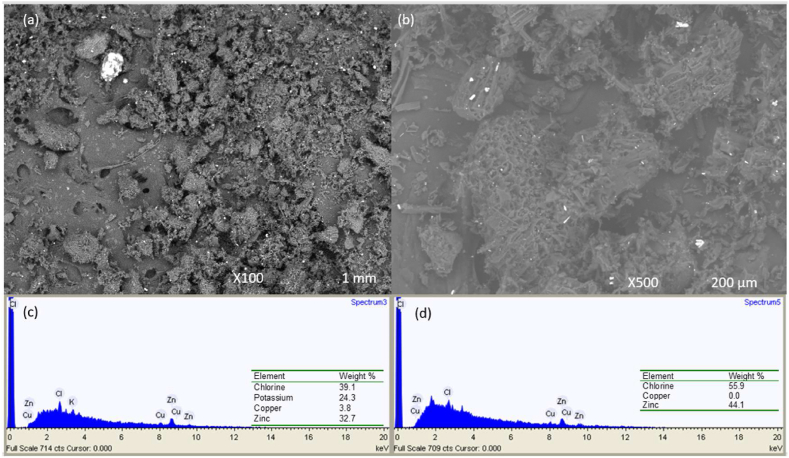
SEM image (a–b) and EDX spectrum (c–d) of the PAC prepared under optimized conditions.

The PJAC morphology before and after Cd^2+^ adsorption is shown using SEM images under different magnification (Fig. 5a–b) and the PAC elemental composition is shown in Fig. 5(c–d). As seen from Fig. 5(a–b). The PJAC surface morphology was rough, porous and had a BET surface area (S_BET_) of 600.4 m^2^/g which provides a relatively substantial number of active sites for Cd^2+^ adsorption. The activated carbon's relative high surface area is because of the presence of many grooves and projections as depicted in Fig. 5(a–b) [49].

**Fig. 6:**
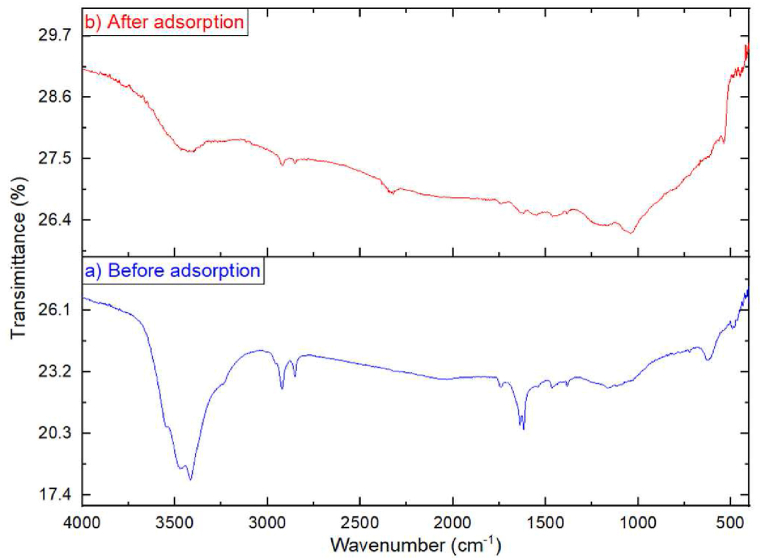
FT-IR spectra of the PJAC prepared under optimal conditions; a) before and b) after adsorption.

#### 3.5.2 FTIR analysis

The characteristic pattern for the adsorption of Cd^2+^ onto both loaded and unloaded PJAC was investigated by using FTIR spectroscopic analysis (Fig. 6). FTIR studies are employed to predict the types of functional groups contained in adsorbent materials [50]. The characteristic FTIR spectra of activated carbon pre and post adsorption provides the presence of preliminary functional groups present in them and which functional groups are engaged in the adsorption process respectively.

Spectra of the sample before adsorption (Fig. 6a) display a wide band within frequency range 3600 to 3000 cm^−1^ which suggests presence of –NH stretching and –OH bonding of hydroxyl, carboxyl group, and phenol or alcohol groups [45]. Peaks of preliminary functional groups of the unloaded adsorbent show a relatively broad peak at 3472 and 3415 cm^−1^ (0-H stretching) [51], sharp peaks at 2921 cm^−1^ and a relatively small peak at 2851 cm^−1^ (C–H stretching alkane) [52] and a presence of a sharp peak at 1618 cm^−1^ (C=C stretching). The study's observed peaks are consistent with those from results found by both [29,34].

After the adsorption of Cd^2+^, the PJAC shows there was reduction in the peak heights and disappearance of some peak heights including some changes in the peak positions of the FTIR spectrum (Fig. 6b). There are significant changes in the position and intensity of peaks in the FTIR spectra after Cd^2+^ adsorption. After adsorption (Fig. 6b), the PJAC shows a significant reduction in peak bands, suggesting that the functional groups are fully engaged in the adsorption process. The observed peak reduction reveals that there was a strong characteristic adsorption of Cd^2+^ onto the PJAC surface. The FTIR study of PJAC shows distinct peaks that indicate availability of various functional groups. These functional groups i.e. methyl, methylene, alkanes, amide, and organo-halogens, are crucial to Cd^2+^ adsorption [53]. Cd^2+^ adsorption has been discovered to be related with Interactions involving carboxylic, hydroxyl and amino acid groups [53,54].

#### 3.5.3 Point of zero charge (pH_PZC_)

**Fig. 7:**
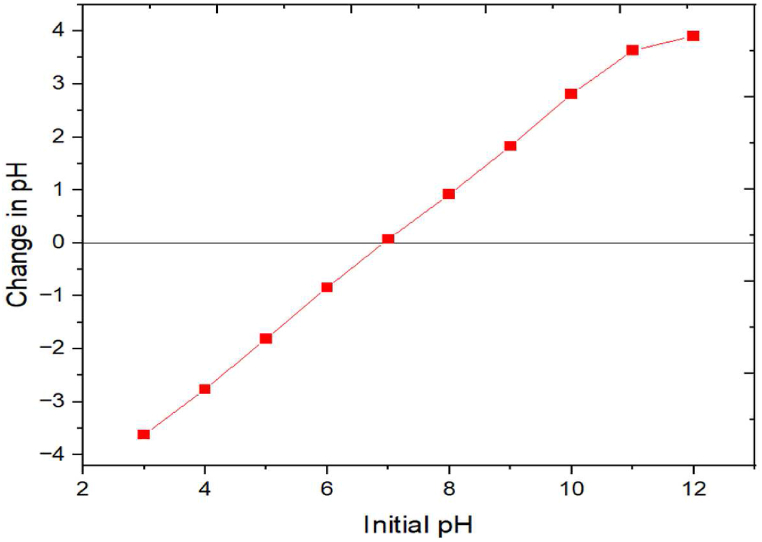
Point of Zero Charge (pH_PZC_) of the prepared PJAC.

Based on precursor source and preparation method, the surface of any activated carbon might be neutral, acidic, or basic. This surface nature of the PJAC in this work was determined by the pH_PZC_ i.e. pH at which the adsorbent surface achieves electrical neutrality and is also referred to as the isoelectric point [55]. In addition, understanding the electrostatic interactions during the adsorption process is contingent upon the pH_PZC_ value. As shown from Fig. 7, the PJAC pH_PZC_ is slightly acidic at 6.92. Several previous studies on the preparation of zinc chloride modified carbons have shown similar slight acidic pH of point of zero charge: 6.85 [56], 6.0 [57] and 5.92 [55].

#### 3.5.4 X-ray diffraction (XRD) analysis

**Fig. 8:**
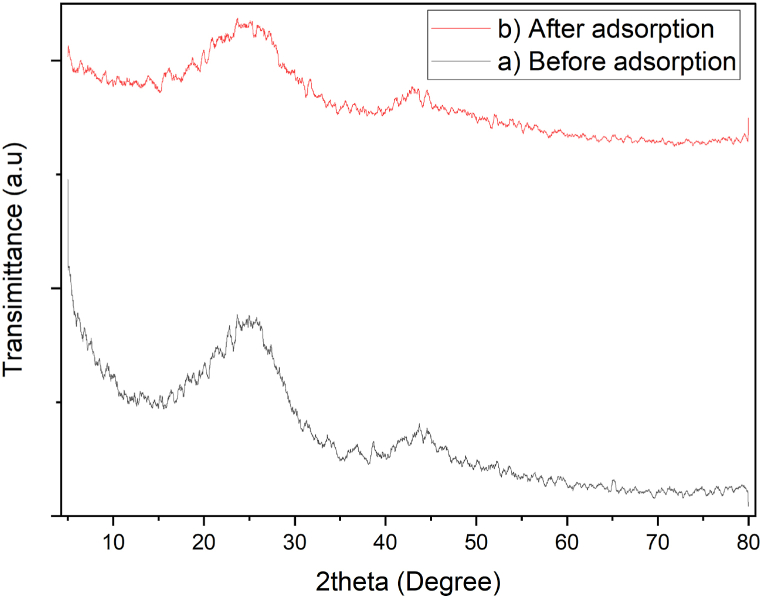
XRD profile for the prepared *Prosopis Juliflora* activated carbon (a)before and (b) after adsorption.

The XRD analysis investigated the amorphous or crystalline structure of the PJAC before (unloaded) and after (loaded) Cd^2+^ adsorption (Fig. 8). From Fig. 8, the presence of high intensity peaks around 23° 2θ angle and low intensity peaks around 44° 2θ angle can be noted which signify the crystalline nature of the synthesized adsorbent. Comparable results have previously been obtained and reported by Refs. [50,53,58,59]. Evidently, the loaded PJAC shows some variations in the intensity of peaks as compared to that of unloaded PJAC due to the adsorption of Cd^2+^ onto PJAC surface.

## 4 Predicted versus experimental Cd^2+^ percentage removal efficiency

With the optimal preparation parameters as shown in Table 5 (i.e. IR = 1.771, T = 595 °C and t = 174 min), new additional activated carbon was freshly prepared and subsequent adsorption experiments in triplicate were run using the same operating conditions as reported in section 2.4 (i.e. Cd^2+^ concentration = 100 mgL^−1^, adsorbate volume = 25 ml, pH = 8, temperature = 25 °C, shaking time = 30 min, PJAC dosage = 0.5 g and shaking speed = 200 rpm) These batch adsorption experiment studies were run for the powdered activated carbon prepared under optimized conditions to compare the predicted against the experimental Cd^2+^ removal efficiencies. An average of 96.7 % Cd^2+^ removal efficiency was experimentally obtained against the 99.9 % removal efficiency that was predicted by the model. The experimental removal efficiency was relatively lower but not significantly different to the predicted Cd^2+^ removal efficiency by adsorption using *Prosopis Juliflora* powder prepared using both chemical and thermal activation.

## 5 Comparative studies

A literature review with other activated carbons prepared from *Prosopis Juliflora* biomass and synthesized in the same way (i.e. chemical and thermal activation) was undertaken to assess the comparative advantage of the synthesized material in this study (Table 6). The S_BET_ of the final PJAC synthesized under optimum conditions formed the basis of the comparison for the main reason that the other papers dealt with other pollutants other than Cadmium used in this study. Table 6 compares the S_BET_ of activated carbon generated from *Prosopis Juliflora* from this study to those previously reported in the literature. The S_BET_ of the activated carbon sythesized in this study was relatively comparable to the S_BET_ of other activated carbon prepared from the same biomass material reported in previous studies (Table 6).

**Table 6:** S_BET_ of activated carbon prepared from *Prosopis Juliflora* reported in previous studies.

Material/Composite	Impregnation Ratio (IR)		Carbonization		Activation		S_BET_ (m^2^/g)	Reference
	Chemical	Ratio	Temp (°C)	Time (min)	Temp (°C)	Time (min)		
Seed/Polyaniline	ZnCl_2_,	–	400	–	800	10	1028	[28]
Wood	KOH	1	600	–	800	–	748.91	[30]
Wood	H_2_SO_4_,	1	500	–	–	–	358.47	[59]
Wood	H_3_PO_4_,	2.5	600	120	–	–	946.06	[60]
Wood	H_2_SO_4_,	1	500	–	–	–	320.49	[34]
Wood	ZnCl_2_	1.8	595	174	800	120	600.4	This Paper

## 6 Conclusion

The BBD was effectively utilized to investigate the influence of three activated carbon preparation factors (chemical impregnation ratio, carbonization temperature, and carbonization time) on the effectiveness of Cd^2+^ removal from an aqueous solution using PJAC. A quadratic model was developed to establish a correlation between the preparation factors and the Cd^2+^ removal efficiency by adsorption. By examining the response surfaces generated by the models, it was shown that the impregnation ratio and carbonization temperature had a notable impact on the removal of Cd^2+^ (p = 0.0003), with carbonization temperature being the most influential factor (p = 0.0001) among the three factors investigated.

Process optimization was conducted and the optimized predicted removal efficiency of 99.9 % with 1.0 desirability was obtained for 1.8 impregnation ratio, 595 °C carbonization temperature and 174 min activation time. Experimentally a removal efficiency of 96.7 % was obtained from the PJAC prepared under optimum conditions. The optimized preparation conditions prepared PJAC was characterized and the SEM images revealed a rough and porous morphological surface with an S_BET_ of 600.4 m^2^/g and a near neutral pH_PZC_ of 6.92. The FTIR spectrum demonstrated a well-defined difference before and after Cd^2+^ adsorption with the latter showing a reduction in both peak intensity and height. The XRD pattern also clearly indicated the crystalline nature of the prepared adsorbent with the loaded activated carbon showing some variations in the intensity of peaks as compared to that of unloaded activated carbon.

## Data availability statement

The data that support the findings of this study are available within the article and/or its supplementary material.

## CRediT authorship contribution statement

**Gilbert C. Chintokoma:** Writing – original draft, Methodology, Investigation, Formal analysis, Conceptualization. **Yonas Chebude:** Writing – review & editing, Validation, Supervision. **Shimelis K. Kassahun:** Writing – review & editing, Supervision.

## Declaration of competing interest

The authors declare that they have no known competing financial interests or personal relationships that could have appeared to influence the work reported in this paper.
